# C3aR1-Deletion Delays Retinal Degeneration in a White-Light Damage Mouse Model

**DOI:** 10.1167/iovs.66.1.15

**Published:** 2025-01-07

**Authors:** Verena Behnke, Anne Wolf, Mandy Hector, Thomas Langmann

**Affiliations:** 1 Laboratory for Experimental Immunology of the Eye, Department of Ophthalmology, Faculty of Medicine and University Hospital Cologne, University of Cologne, Cologne, Germany; 2 Center for Molecular Medicine Cologne (CMMC), Cologne, Germany

**Keywords:** microglia, light damage, complement, anaphylatoxin receptors, age-related macular degeneration (AMD)

## Abstract

**Purpose:**

In the aging retina, persistent activation of microglia is known to play a key role in retinal degenerative diseases like age-related macular degeneration (AMD). Furthermore, dysregulation of the alternative complement pathway is generally accepted as the main driver for AMD disease progression and microglia are important producers of local complement and are equipped with complement receptors themselves. Here, we investigate the involvement of anaphylatoxin signaling, predominantly on Iba1^+^ cell activity, in light-induced retinal degeneration as a model for dry AMD, using anaphylatoxin receptor knockout (KO) mice.

**Methods:**

Bright white light with an intensity of 10,000 lux was applied for 30 minutes to complement component 3a receptor 1 (*C3ar1*) or complement component 5a receptor 1 (*C5ar1*) KO and wildtype (WT) mice. Analyses of transcriptome changes and migration activity of Iba1^+^ cells as well as retinal thickness were performed 4 days after light exposure.

**Results:**

Full body KO mice of either C3aR1 or C5aR1 were tested, but none led to mitigated migration of Iba1^+^ cells to the subretinal space or decreased expression of complement factors after light damage compared to WT mice. However, a partial rescue of retinal thickness was shown in C3aR1 KO mice, which was mirrored by significant less membrane attack complex (MAC) occurrence in the outer retina.

**Conclusions:**

We conclude that deletion of the anaphylatoxin receptor C3aR1 cannot modulate mononuclear phagocytes but diminishes retinal degeneration through interference with the complement pathway and thus decreased MAC assembling. C3aR1-targeted therapy may be considered for patients with dry AMD.

A variety of complement proteins, receptors, and regulators are expressed in the retina.^1^ The cleavage of C3 and C5 by their respective convertases are the central steps in all complement pathways and generate toxic complement proteins C3a and C5a, respectively. Both so-called anaphylatoxins drive inflammation by engaging their corresponding chemotactic receptors C3aR1 and C5aR1 (also named CD88),^2^^–^^4^ which are primarily expressed on myeloid and lymphoid cells throughout the body.^5^^–^^10^ Anaphylatoxins are the most potent pro-inflammatory fragments generated during the complement pathway and function as chemoattractants for mononuclear phagocytes.^11^^,^^12^

Hence, microglia are, alongside the RPE, important producers of local complement and are equipped with complement receptors themselves.^13^ Microglia are the resident immune cells of the central nervous system.^14^ In homeostasis, they build a regularly spaced network of ramified cells in the plexiform layers,^15^^,^^16^ whereas, in the aging retina, persistent activation of microglia is known to play a key role in retinal degenerative diseases like age-related macular degeneration (AMD).^17^^–^^19^

AMD is a major blinding disease in the elderly of the western world, with the number of affected people rising rapidly. In terminal stages of its two forms, dry and wet, AMD can lead to complete vision loss that severely decreases the patient's quality of life. Representing a multifactorial disease, main risk factors for AMD are advanced age, smoking, and variations in genes of the immune system, especially those regulating the complement system.^20^^,^^21^ Although numerous studies raise convincing evidence that the overly activated complement system in retinas can lead to AMD and a variety of complement regulators have been generated and tested in clinical studies,^22^ effective treatment options are lacking. Here, we investigate the involvement of anaphylatoxin signaling, predominantly on Iba1^+^ cell activity, during retinal degeneration using anaphylatoxin receptor knockout (KO) mice.

## Materials and Methods

### Cell Culture

Murine BV-2 cells^23^ were cultured in T75 flasks at 37°C and 5% CO_2_ humidity. RPMI 1640 (Gibco, Waltham, MA, USA) culture medium contained 5% fetal bovine serum (FBS; Gibco, Waltham, MA, USA), 1% penicillin/streptomycin (Gibco, Waltham, MA, USA), 3 mM L-glutamin (Gibco, Waltham, MA, USA), and 50 µM β-Mercaptoethanol (Sigma-Aldrich, Darmstadt, Germany). Media was changed every 3 days and cells were split at 95% confluency. BV-2 cells were cultured in 6-well plates, with 3*10^5^ cells per well and treated with 50 ng/mL lipopolysaccharide (LPS; InvivoGen, San Diego, CA, USA) for 6, 24, or 72 hours.

### Animals

All experimental protocols complied with the ARRIVE guidelines and were carried out in accordance to the German Animal Welfare Act, which is in line with the European Directive 2010/63/EU, and the ARVO Statement for the Use of Animals in Ophthalmic and Vision Research. The animal experiments used in this study were reviewed and approved by the governmental body responsible for animal welfare in the state of North Rhine-Westphalia, Germany (Landesamt für Natur, Umwelt, und Verbraucherschutz).

BALB/cJRj mice were purchased from Janvier. C.129S4-*C3ar1^tm1Cge^*/J and C.129S4(B6)-*C5ar1^tm1Cge^*/J, further referred to as *C3ar1* and *C5ar1*, were purchased from Jackson Laboratory (stock number 005712 and 006845, respectively), backcrossed two times with BALB/cJ and bred in-house. The animals were kept under SPF-conditions in an air-conditioned environment with a 12-hour light/dark cycle and water and food ad libitum. Eight to 10-week-old male and female BALB/cJ mice were used in the experiments.^24^^–^^26^ For genotyping, DNA was isolated from ear punches via the HotSHOT Method.^27^ PCR was performed based on Jackson Laboratories’ protocols with the Taq-S PCR kit (Genaxxon). The primers used were as follows: *C3ar1*, forward common 5′-agccattctaggggcgtatt-3′, reverse wildtype (WT) 5′-tggggttatttcgtcttctgc-3′, reverse mutant 5′-tggatgtggaatgtgtgcgag-3′; *C5ar1*, forward WT 5′-ggtctctccccagcatcata-3′, forward mutant 5′-gccagaggccacttgtgtag-3′, and reverse common 5′-ggcaacgtagccaagaaaaa-3′. C3ar1 and C5ar1 PCR products were separated on a 2% / 1% (w/v) agarose gel regarding their size (WT = 250 bp and KO = 400 bp and WT = 386 bp and KO = 244 bp), respectively.

### Light Exposure

Littermates were dark-adapted for 16 hours prior to light exposure. The pupils were dilated with 2.5% phenylephrine and 1% tropicamide under dim red light and the mice were placed separately or in pairs in reflective, aluminum-foil-coated cages to prevent covering. Bright white light with an intensity of 10,000 lux for 30 minutes was applied. After light exposure, the animals were transferred to normal light cycle until further analysis.

### In Vivo Imaging Using Spectral-Domain Optical Coherence Tomography and BluePeak Autofluorescence 

Retinal thickness using spectral domain optical coherence tomography (SD-OCT) and BluePeak autofluorescence (BAF) to investigate structural changes in the retina were performed on both eyes 4 days after light exposure with the Spectralis HRA + OCT device (Heidelberg, Germany). The mice were anesthetized with a mixture of ketamine (100 mg/kg body weight, Ketavet; Pfizer Animal Health) and xylazine (5 mg/kg body weight, 2% Rompun; Bayer HealthCare) diluted in 0.9% sodium chloride by intraperitoneal (IP) injection and their pupils were dilated with a topical drop of phenylephrine 2.5%–tropicamide 0.5% before image acquisition. Retinal thickness measurements were performed using the Heidelberg Eye Explorer Software using a circular ring scan (circle diameters 3 and 6 mm), centered on the optic nerve head, which represents the average retinal thickness (µm) of each quadruplet.

### RNA Isolation, Reverse Transcription, and Quantitative Real-Time Polymerase Chain Reaction 

RNA from cultured BV-2 microglia cells was isolated with the RNA Isolation Kit following the manufacturer's instructions (Machery & Nagel, Düren, Germany). RNA from retinas was isolated with the Qiagen Micro isolation kit according to the manufacturer’s protocol. Purity and integrity of the RNA was assessed with a NANODrop 2000 machine (Thermo Fisher Scientific, Waltham, MA, USA). The cDNA was synthesized with the Thermo Fischer Reverse Transcriptase Kit according to the company's protocol (Thermo Fisher Scientific, Waltham, MA, USA). Subsequent quantitative real-time PCR (qRT-PCR) analysis was performed in duplicates with either the Takyon Probe Assay protocol (Eurogentec Deutschland GmbH, Cologne, Germany) or Takyon SYBR Green Assay protocol using the LightCycler 480 II (Roche, Basel, Switzerland). Primer sequences and Roche Universal Probe Library probe numbers for probe-based assay were as follows ATP synthase, H^+^-transporting, mitochondrial F1 complex, β polypeptide (Atp5b), forward primer 5′-ggcacaatgcaggaaagg-3′, reverse primer 5′-tcagcaggcacatagatagcc-3′, probe #77; Complement C1q A chain (C1qa), forward primer 5′-ggagcatccagtttgatcg-3′, reverse primer 5′-catccctgagaggtctccat-3′, probe #16; Complement component 3 (C3), forward primer 5′-accttacctcggcaagtttct-3′, reverse primer 5′-ttgtagagctgctggtcagg-3′, probe #76; complement component 6 (C6), forward primer 5′-AAGGAAGACACGTGCACCAA-3′, reverse primer 5′- TCCTTCACCGATTCTAGCCAC -3; complement component 7 (C7), forward primer 5′-GGTGTGCTTTATAGCAGCGTT-3′, reverse primer 5′-CTGAACGCCTTCGAGTCTGAG-3; complement component 8a (C8a), forward primer 5′-AAACGCCACCTGGTGTGTAA-3′, reverse primer 5′-AGGATGTTGTACCCCAAGGC-3; complement component 9 (C9), forward primer 5′-CAGCAGGCTATGGGATCAACA-3′, reverse primer 5′-CGGTCACAGAGTCCGTTGTA-3; complement factor b (Cfb) forward primer 5′-ctcgaacctgcagatccac-3′, reverse primer 5′-tcaaagtcctgcggtcgt-3′, probe #1; complement factor h (Cfh), forward primer 5′-gaaaaaccaaagtgccgaga-3′, reverse primer 5′-ggaggtgatgtctccattgtc-3′, probe #25; inducible nitric oxide synthase (iNos), forward primer 5′-ctttgccacggacgagac-3′, reverse primer 5′-tcattgtactctgagggctga-3′, probe #13; Translocator protein (Tspo), forward primer 5′-cccttgggtctctacactgg-3′, reverse primer 5′-aagcagaagatcggccaag-3′, probe #21. ATPase was used as reference gene and the ΔΔC method was applied using the LightCycler 480 software 1.2.1 for data evaluation.

### Immunofluorescence Staining

Eyes were enucleated and fixed in 4% paraformaldehyde (Roti Histofix; Roth, Karlsruhe, Germany) for 2 hours at room temperature. For retinal flat mounts, eyes were dissected and incubated with PERM/Block Buffer (5% NDS, 0.2% BSA, and 0.3% Triton X-100 in PBS) overnight at 4°C. For cryosections, eyes were transferred in 10%, 20%, and 30% sucrose for 1 hour each before embedding in optimal cutting temperature compound. For each eye, 10-µm sections were prepared with a Leica CM3050 S Cryostat (Leica Biosystems, Wetzlar, Germany). Frozen slides were thawed at room temperature, dehydrated in PBS, and unspecific antigens were blocked with BLOTTO (1% milk powder and 0.3% Triton X-100 in PBS) for 30 minutes at room temperature. Retinal flat mounts and sections were incubated with primary antibody overnight at 4°C. A 1:500 dilution of anti-ionized calcium-binding adapter molecule 1 (Iba1) antibody (FUJIFILM Wako Chemicals Europe, Neuss, Germany) or C5b-9 (Abcam, Cambridge, United Kingdom) was used. Afterward, the tissue was incubated with anti-rabbit Alexa Fluor 488/ 647 (1:1000) secondary antibody for 1 hour at room temperature (Invitrogen, Carlsbad, CA, USA). Retinal flat mounts were mounted on microscopic slides and embedded with Vectashield H-1400 (Vector Laboratories, Burlingame, CA, USA). Sections were mounted in Fluoromount-G with Dapi (Thermo Fisher Scientific, Waltham, MA, USA).

### TUNEL Assay

Retinal cryosections were labeled with an in situ cell death detection kit RED (Roche) according to the manufacturer's instructions. Fluoromount-G with DAPI was used to counterstain the nuclei in mounted sections.

### Image Analysis

Images of the central retina were taken with a Zeiss Imager M.2 equipped with ApoTome.2 (Oberkochen, Germany). Autofluorescence was recorded at 594 nm in the red channel. The total number of Iba1^+^ cells was counted in four images of each retinal flat mount or five sections and averaged for one retinal n. TUNEL assay images were analyzed by counting the TUNEL^+^ cells and the number of photoreceptor nuclei in the outer nuclear layer (ONL). The percentage of TUNEL^+^ cells in the ONL was calculated as the ratio of TUNEL^+^ nuclei to the total number of photoreceptor nuclei in the ONL, multiplied by 100. Counting was performed with the particle analyzer plugin or the multi-point tool of ImageJ version 1.52a (Bethesda, MD, USA).

### Enzyme-Linked Immunosorbent Assay

The concentration of cytokines in total retinal lysates were measured by ELISA. Tissue samples were sonicated in 1× PBS supplemented with protease and phosphatase inhibitors (complete protease inhibitor cocktail; Roche). The CCL2 ELISA Kit was purchased from R&D Systems and performed according to the manufacturer’s protocol. Absorbance was measured with a TECAN infinite M1000.

### Statistical Analysis

All data were plotted and analyzed with GraphPad PRISM version 7.04. After the D’Agostino & Pearson normality tests, the data were analyzed using 1-way ANOVA followed by Tukey’s multiple comparison post-test. Comparisons between all groups were made, however, if only two groups were compared in one experiment the Mann Whitney *t*-test was performed as indicated (no asterisk: not significant, *P < 0.05, **P < 0.01, ***P ≤ 0.001, and ****P < 0.0001). Error bars show mean ± SEM.

## Results

Anaphylatoxins C3a and C5a drive inflammation by engaging their corresponding chemotactic receptors C3aR1 and C5aR1^2^^–^^4^ and attract mononuclear phagocytes as a consequence.^11^^,^^12^ Anaphylatoxin receptors were found significantly upregulated in LPS-treated BV-2 microglia cells and retinal lysates of BALB/c wildtype mice after light damage (Supplementary Fig. S1). Therefore, we used anaphylatoxin receptor KO mice to elucidate the involvement of anaphylatoxin signaling during retinal degeneration with regard to mononuclear phagocyte activity, with microglia being the resident immune cells of the retina.^14^ For this, 8 to 10-week-old WT or KO animals, determined using PCR (Supplementary Figs. S2A, S2B), of mouse lines *C3ar1* and *C5ar1* with BALB/cJ background, were dark adapted for 16 hours before light exposure to 10,000 lux for 30 minutes (Supplementary Fig. S2C). In the mouse model of light exposure, monocytes are known to infiltrate the retina from the blood stream and mix with residential microglia.^28^ Hence, we will refer to the Iba1^+^ population in the retina after light exposure as mononuclear phagocytes. All analysis assessed below were performed 4 days after light exposure.

### C3ar1 KO Mice Tend to a More Regulated Alternative Complement Pathway Gene Expression

**Figure 1.**
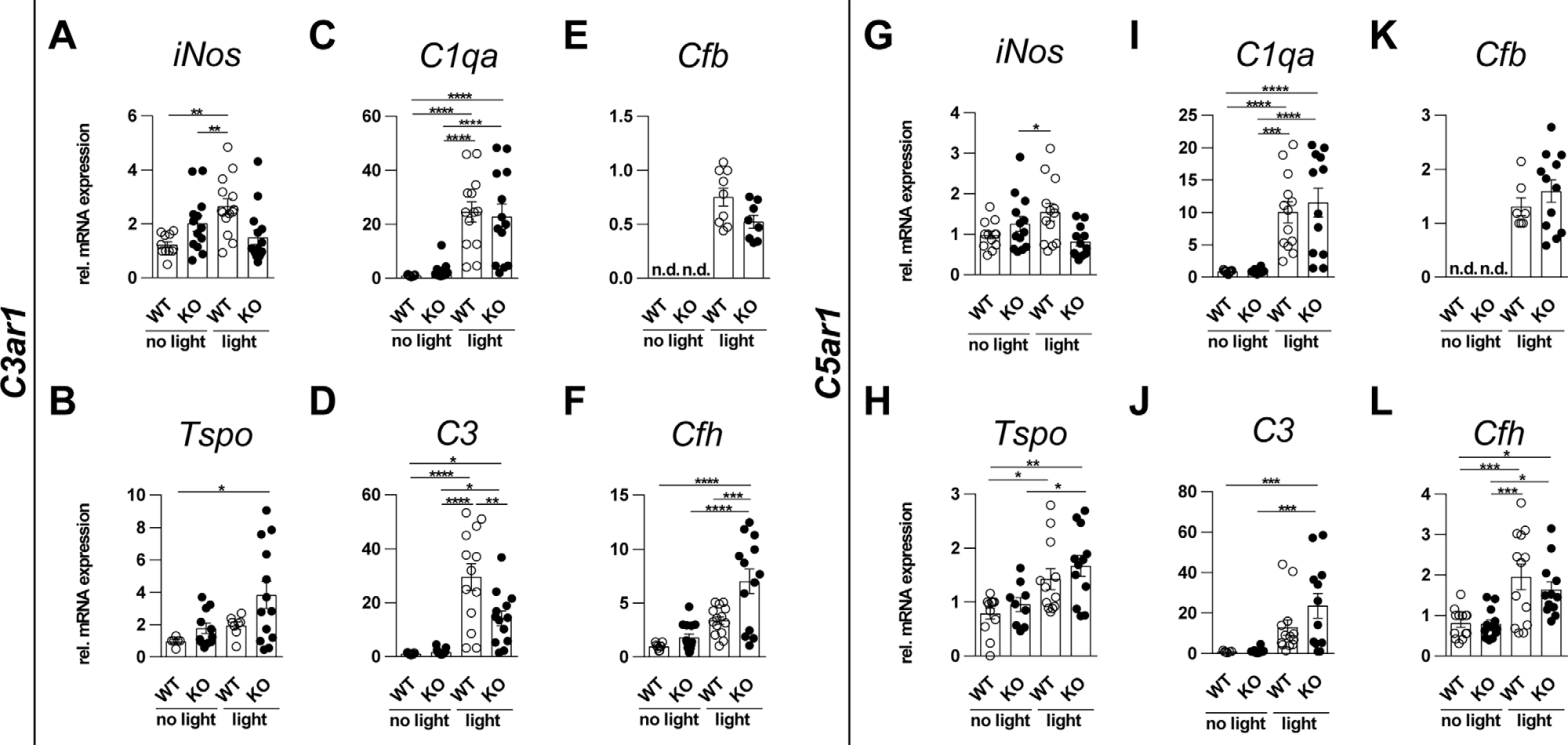
Expression analysis of retinal tissue. Retinas were analyzed 4 days after light damage with 10,000 lux for 30 minutes. The qRT-PCR was performed and ΔΔCT analysis was used for quantification. ATP5B was used as reference gene. Graphs were plotted with GraphPad Prism version 7.04. Bars represent mean ± SEM. Data were analyzed using 1-way ANOVA followed by Tukey’s multiple comparison post-test (*n* = WT, KO, light-damaged WT, and light-damaged KO; C3ar1: *n* = 11, 15, 13–14, 13; TSPO: 6, 11, 8, 13; CFB: 0, 0, 9, 8; C5aR: *n* = 12–13, 12, 13, 12–13, TSPO: 12, 9, 12, 12; and CFB: 0, 0, 7, 12).

To examine transcription changes of complement components and inflammation modulators, gene expression analysis via qRT-PCR of retinal tissue was performed (Fig. 1, Supplementary Fig. S4) Technical duplicates of one retinal expression measurement were averaged for one n. Four days after light exposure, the transcripts of the inflammation marker *iNos* and the early mononuclear phagocyte activation marker *Tspo* remained mostly unchanged (see Figs. 1A, 1B, 1G, 1H), with the exceptions of *iNos* in light-damaged *C3ar1* and *C5ar1* WT mice as well as *Tspo* in *C5ar1* KO mice after light damage (see Figs. 1A, 1G, 1H). In the matter of the complement system, light damage significantly upregulated *C3* and *C1qa* transcripts regardless of the genotype (see Figs. 1C, 1D, 1I, 1J). *Cfb* transcripts could not be detected in untreated samples; thus, data were normalized to light-exposed WT samples (see Figs. 1E, 1K). *Cfb* transcripts were unaltered between light-exposed WT and KO mice. Transcripts of regulatory *Cfh* displayed significant elevation due to light exposure (see Figs. 1F, 1L). *C7* was the only terminal component found significantly upregulated in *C3ar1* WT retinas (see Supplementary Figs. S4A–D), whereas In *C5ar1* animals, *C6*, *C7*, and *C8a* transcripts were significantly enhanced in KO mice (see Supplementary Figs. S4E–H). These data indicate that light damage induces complement signaling accompanied by light inflammation in the retina, with tendencies of lower induction of central factor *C3* and *Cfb* as well as higher levels of regulatory *Cfh* in C3aR1 KO mice compared to WT mice.

### Genetic Ablation of C3ar1 or C5ar1 Does Not Change Microgliosis

**Figure 2.**
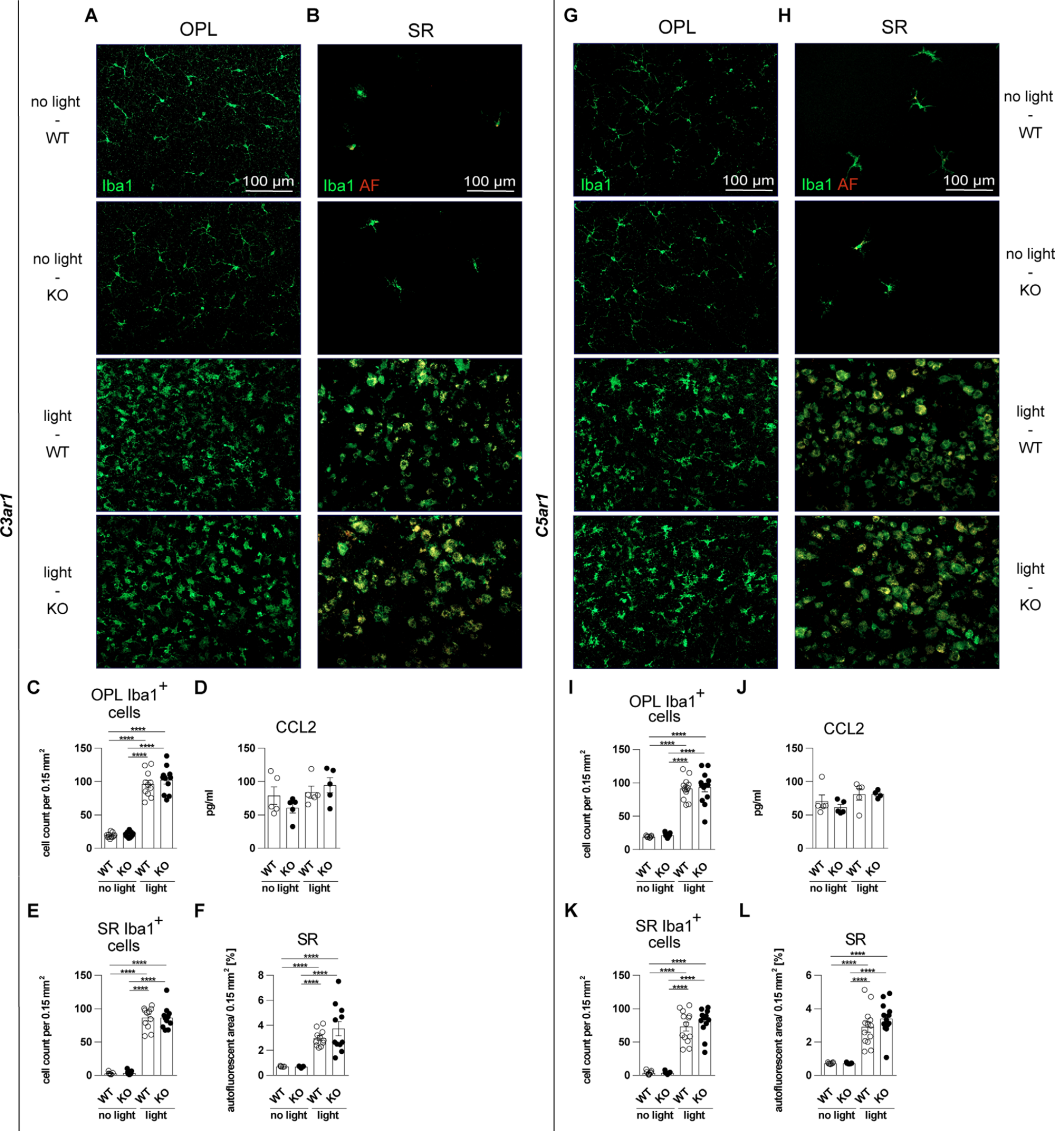
Morphological and migration analysis of microglia in the retina. Retinas were analyzed 4 days after light damage with 10,000 lux for 30 minutes. (**A**, **B**, **G**, **H**) Mononuclear phagocytes were stained on retinal flat mounts against ionized calcium-binding adapter molecule 1 (Iba1). (**C**, **E**, **F**, **I**, **K**, **L**) Four images of the central retina were taken around the optic nerve of each eye and averaged for 1 n. Cell numbers were counted using the particle analyzer plugin of ImageJ. (*C3ar1*: *n* = 11 and *C5ar1 n* = 13). (**D**, **J**) ELISA was performed with full retinal lysates (*n* = 5, *C5ar1* light damage KO: *n* = 4). Data were analyzed using 1-way ANOVA followed by Tukey’s multiple comparison post-test. Graphs were plotted with GraphPad Prism version 7.04. Bars represent mean ± SEM. AF, autofluorescence; CCL2, CC-chemokine ligand 2; OPL, outer plexiform layer; SR, subretinal space.

**Figure 3.**
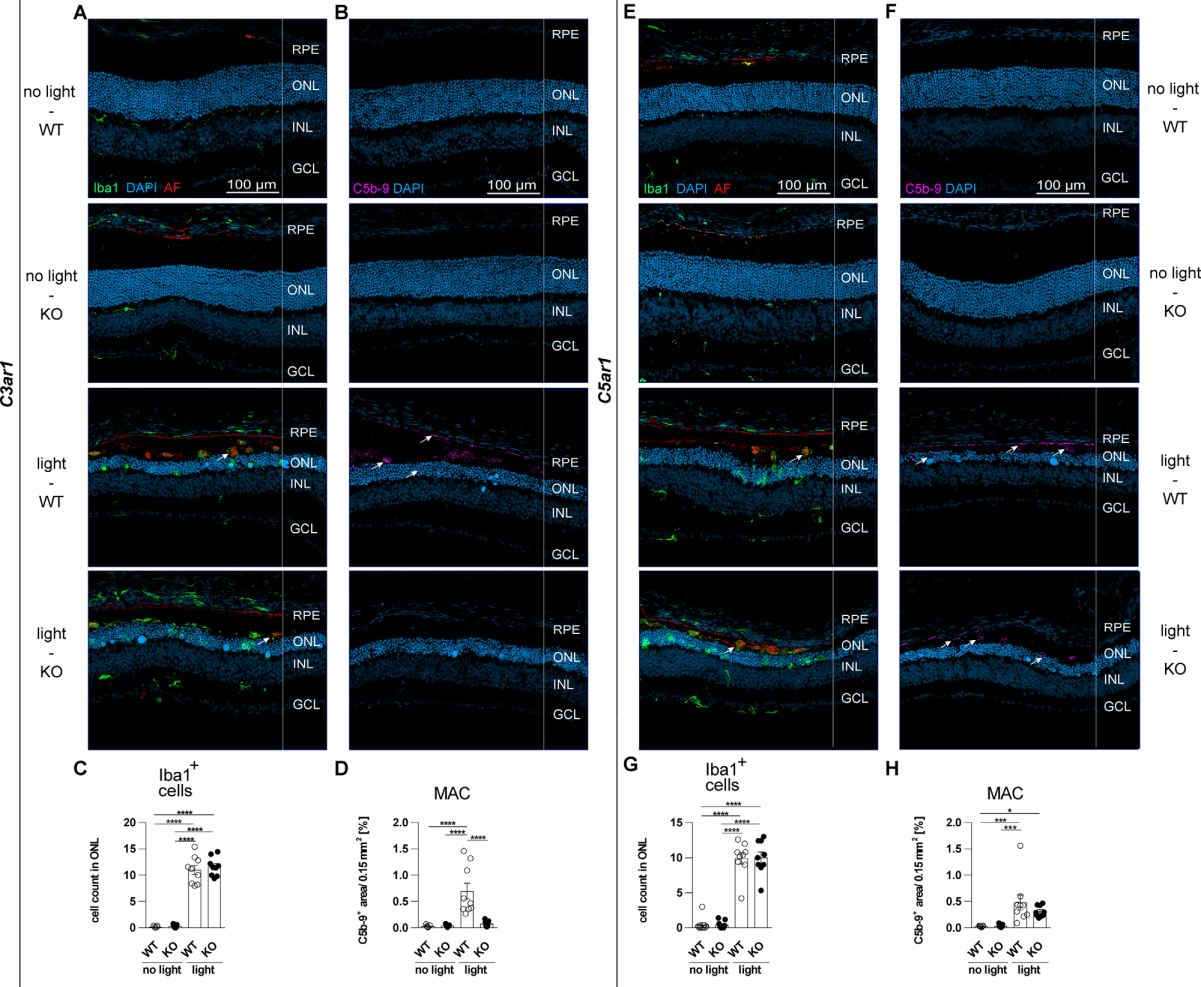
Spatial analysis of microglia and complement membrane attack complex (MAC) in the eyes. The eyes were analyzed 4 days after light damage with 10,000 lux for 30 minutes. (**A**, **E**) Cryosections were stained with DAPI and against ionized calcium-binding adapter molecule 1 (Iba1). (**B**, **F**) Cryosections were stained with DAPI and against MAC (C5b-9). (**C**, **D**, **G**, **H**) Cell numbers were counted using the multi-point tool of ImageJ. Five images of the central retina were taken of each eye and averaged for 1 n (*n* = 9). Data were analyzed using 1-way ANOVA followed by Tukey’s multiple comparison post-test. Graphs were plotted with GraphPad Prism version 7.04. Bars represent mean ± SEM. AF, autofluorescence; GCL, ganglion cell layer; INL, inner nuclear layer; OPL, outer plexiform layer; ONL, outer nuclear layer; RPE, retinal pigment epithelium; SR, subretinal space.

Systematic morphometric analysis of mononuclear phagocytes using Iba1-stained retinal flat mounts and cryosections revealed no change in microgliosis among light-challenged genotypes (Figs. 2, 3). An evenly spaced network of ramified microglia was evident in the outer plexiform layer (OPL) of unchallenged mice (see Figs. 2A, 2G), which significantly increased after light damage (see Figs. 2C, 2I). Furthermore, light-challenged mononuclear phagocytes showed a more amoeboid shape with some rudiment protrusions. Untreated controls depicted very few activated microglia in the subretinal space (SR), whereas light damage significantly increased these numbers of migrated mononuclear phagocytes (see Figs. 2B, 2H). These data were further supported by CC-chemokine ligand 2 (CCL2) protein levels in the retina (see Figs. 2D, 2J). Concurrent to mononuclear phagocyte activation and migration, these cells became amoeboid and enhanced their phagocytic reactivity, shown by increased autofluorescence co-localized with subretinal mononuclear phagocytes (see Figs. 2B, 2H lower panels, see Supplementary Fig. S3). Nevertheless, there were no differences in mononuclear phagocyte numbers and autofluorescent area found in the SR of light-challenged retinas (see Figs. 2E, 2F, 2K, 2L). Iba1-stained cryosections confirmed the results from retinal flat mounts, that phagocytosed autofluorescent material co-localized with subretinal mononuclear phagocytes (see Figs. 3A, 3E; white arrows). Shredded photoreceptor disks and byproducts of the visual cycle are recycled in the RPE,^29^^,^^30^ which was visible as autofluorescent material in the RPE cell layer. Counting of mononuclear phagocytes in the ONL revealed migration after light damage in both WT and KO animals (see Figs. 3C, 3G).

### C3aR1 KO Leads to Decreased MAC Formation

Membrane attack complex (MAC; C5b-9) formation represents the terminal pathway of the complement cascade and either leads to pore formation on the cell surface and cell lysis^31^ or induces intracellular inflammatory signaling.^32^^,^^33^ Expression level analysis indicated lower terminal expression levels of terminal complement components in KO mice compared to WT mice (see Supplementary Figs. S4A–S4D). Cryosections stained for C5b-9 revealed increased MAC formation in the outer retina of light-challenged mice (see Figs. 3B, 3F; white arrows). Interestingly, significantly less MAC was detected in C3aR1 KO mice than WT mice (see Fig. 3D).

### C3aR1-Deficiency Delays Retinal Thinning

**Figure 4.**
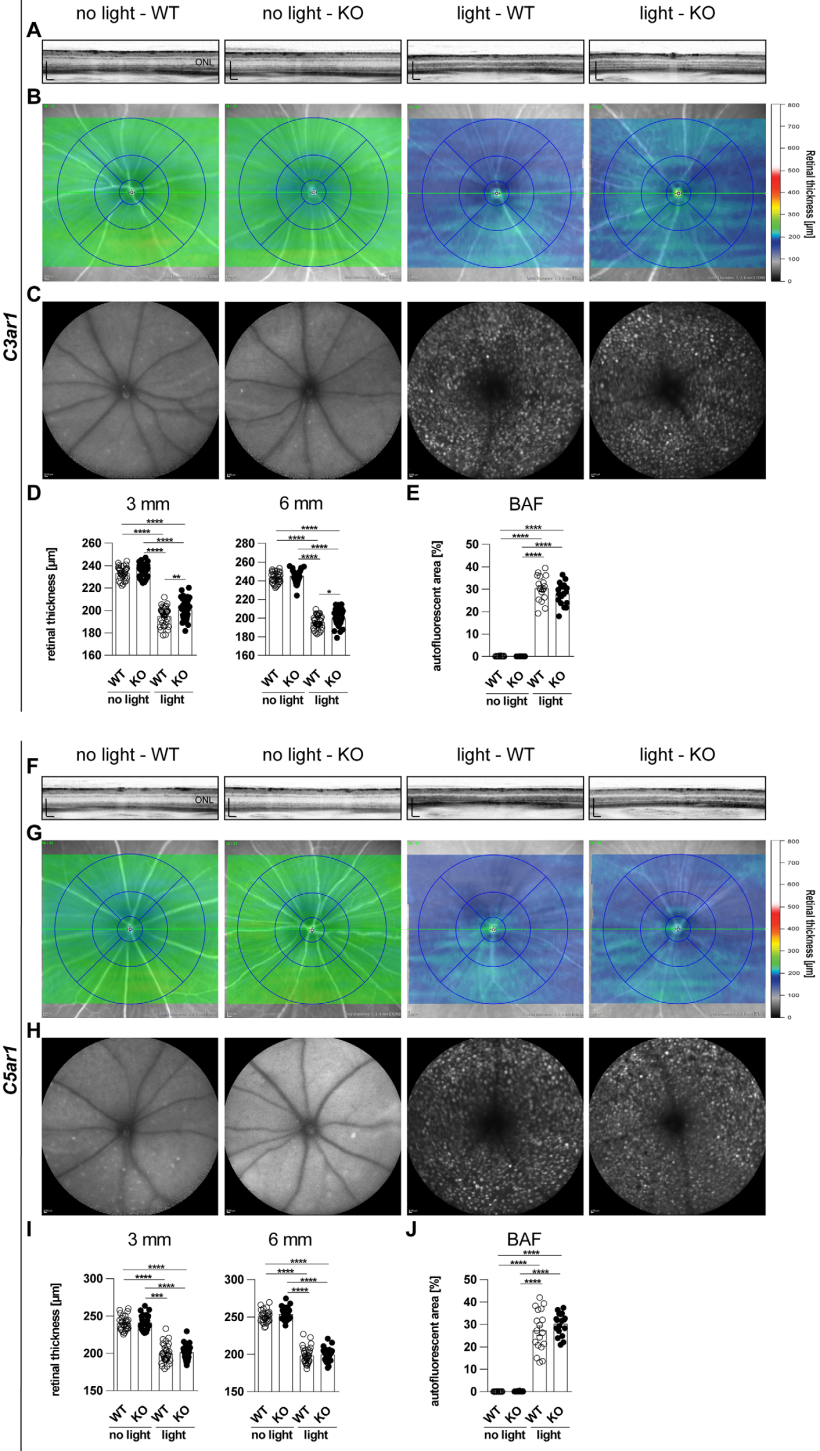
Retinal thickness and OCT analysis. Eyes were analyzed 4 days after light damage with 10,000 lux for 30 minutes. (**A**, **F**) Representative scans and (**B**, **G**) corresponding heatmaps compiled by spectral-domain optical coherence tomography (SD-OCT) displayed retinal overviews. (**C**) BluePeak autofluorescence (BAF) images depict retinal background. (**D**, **I**) Retinal thickness was assessed in circular ring scans (circle diameters 3 and 6 mm), centered on the optic nerve head, which represents the average retinal thickness (µm) of each quadruplet. (C3aRr1: *n* = 36 and C5ar1: *n* = 32). (**E**, **J**) Percentage of autofluorescent area was measured with ImageJ with the same region of interest for all images (*n* = 18). Data were analyzed using 1-way ANOVA and Tukey's multiple comparison test. Graphs were plotted with GraphPad Prism version 7.04. Bars represent mean ± SEM.

As DAPI-staining in cryosections already indicated light damage-induced thinning of the outer nuclear layer, we quantified the overall retinal thickness using SD-OCT scans (Fig. 4). Here, OCT scans confirmed thinning of the ONL with light damage, which changed the overall reflectance patterns of the retina (see Figs. 4A, 4F). As clearly indicated by color changes in retinal heatmaps (see Figs. 4B, 4G), analysis revealed a significant reduction of retinal thickness after light damage (see Figs. 4D, 4I). However, the retinal thickness of light-challenged C3aR1 KO animals significantly exceeded that of WT mice (see Fig. 4D).

### Genetic Anaphylatoxin Receptor Ablation Does Not Alter Autofluorescent Material Accumulation

Accumulation of autofluorescent material is a hallmark of AMD disease progression. Hyper-reflective foci appear as drusen in retinal fundus images, which are already detectable in patients with intermediate AMD.^34^ Complement components like Anaphylatoxins are prevalent components of drusen^12^^,^^35^^–^^39^; therefore, BAF images were integrated in the study (see Figs. 4C, 4H). The autofluorescent area in fundus images was significantly increased after light damage, however, unaltered among genotypes (see Figs. 4E, 4J).

### Similar Cell Death in C3aR1 KO and WT Mice Retinas

**Figure 5.**
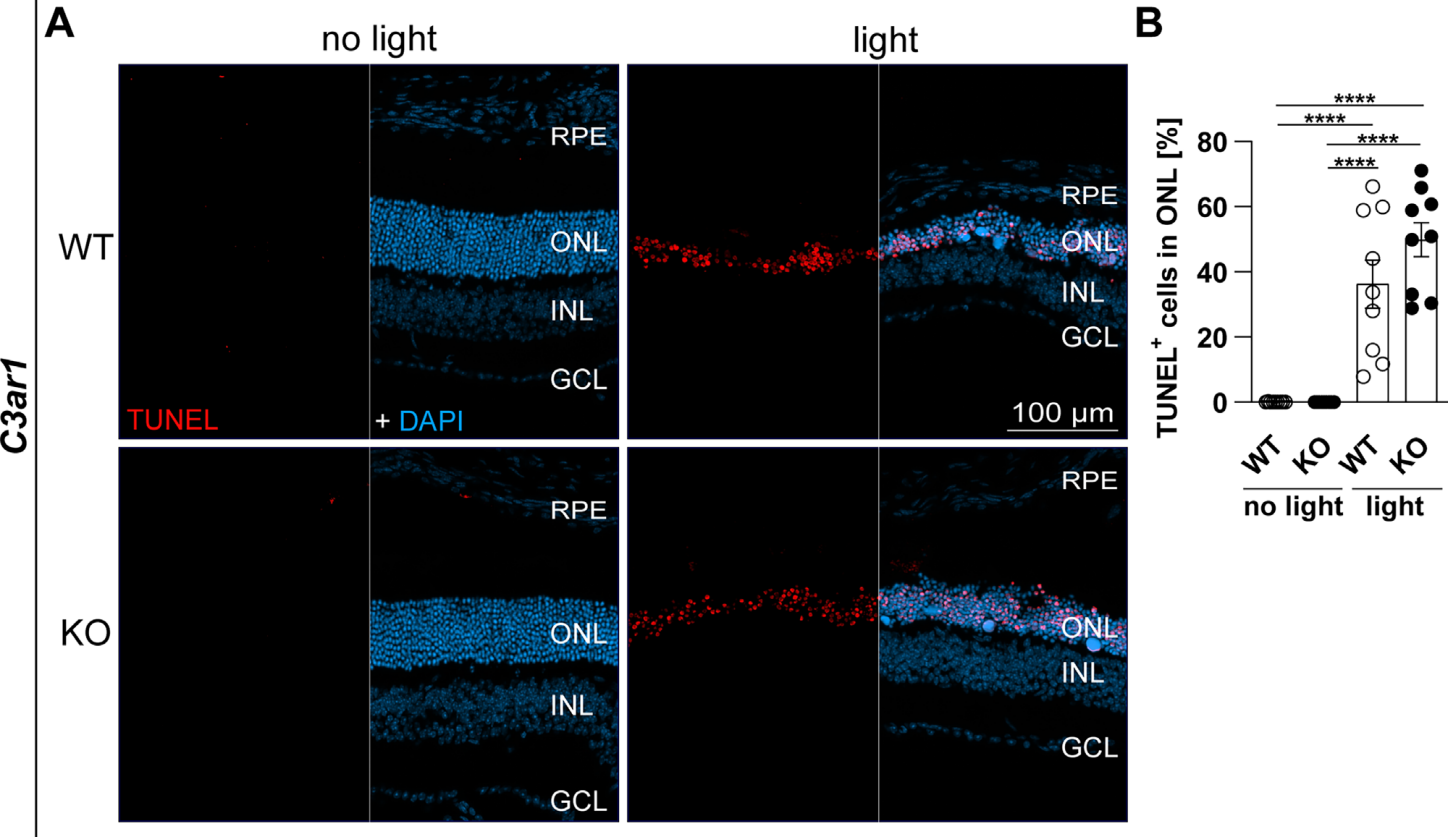
Apoptosis of photoreceptors in the outer nuclear layer. Eyes were analyzed 4 days after light damage with 10,000 lux for 30 minutes. (**A**) Apoptotic cells on cryosections were labeled with TUNEL assay and counterstained with DAPI. (**B**) Cell numbers were counted using the particle analyzer plugin of ImageJ in outer nuclear layer (ONL) specific ROIs, set individually for each image via DAPI staining. Five images of the central retina were taken of each eye and averaged for 1 n (*n* = 9). Data were analyzed using 1-way ANOVA followed by Tukey’s multiple comparison post-test. Graphs were plotted with GraphPad Prism version 7.04. Bars represent mean ± SEM. GCL, ganglion cell layer; INL, inner nuclear layer; ONL, outer nuclear layer; RPE, retinal pigment epithelium.

OCT scans revealed that retinal thickness of light-challenged C3aR1 KO animals significantly exceeded that of WT mice. In addition, in situ retinal cell death was detected and quantified in cryosections of *C3ar1* mice via TUNEL (Fig. 5). Following light damage, TUNEL^+^ cells were visible in the ONL (see Fig. 5A), without significant changes between WT and KO mice (see Fig. 5B).

## Discussion

Anaphylatoxins are potent inflammatory mediators, therefore are implicated in inflammatory diseases.^40^ C3a and C5a drive inflammation by engaging their corresponding chemotactic receptors C3aR1 and C5aR1, respectively.^2^^–^^4^ Thus, full body KO mice of either *C3ar1* or *C5ar1* were tested in an experimental mouse model mimicking some features of dry AMD. Here, we showed that none of the KOs led to mitigated mononuclear phagocyte migration to the subretinal space, albeit C3aR1 KO decelerated retinal thinning.

Dysregulation of the alternative complement pathway is generally accepted as a main driver for AMD disease progression. Despite numerous experimental mouse models and clinical studies focused on complement inhibition in AMD, effective treatment options are still lacking. Although anaphylatoxin receptors C3aR1 and C5aR1 were found to be highly expressed on immune cells after light exposure, reduced microgliosis could only be detected in C5aR1-deficient mice, but not C3aR1-deficient mice, and neither of the KO mice were able to preserve retinal thickness after light exposure.^41^ In line with this, in another study, C3aR1 KO and C3aR1/C5aR1-double KO mice, but not C5aR1 KO mice, had progressive retinal cell loss and dysfunction after light damage.^4^ However, there is still a debate if one anaphylatoxin signaling axis is pro-inflammatory and the other one is protective or if both are mandatory for retinal function and structure. Our results observed no change in mononuclear phagocyte activity in the outer retina and only a partial rescue of the retinal thickness in C3aR1-deficient mice. Indeed, *C3ar1*, but not *C5ar1*, was found upregulated in BALB/c animals in our study (see Supplementary Fig. S1). All studies mentioned before used BALB/cJ mice, but Yu et al. furthermore compared their results with C57BL/6J mice and could not report differences, eliminating potential influences of the genetic background.^4^ However, major experimental settings as light regime and analysis time points as well as age and sex of mice differed among all studies. Noteworthy, while often termed a decoy receptor, pro-inflammatory functions have been proposed for the second C5a ligand C5aR2.^42^ Antibody blockade or targeted deletion of C5aR2 resulted in excessive C5a-mediated chemotaxis,^43^^,^^44^ suggesting the absence of C5aR1 in our model prohibits heterodimerization and therefore may alter C5aR2 function. Despite the fact that most pro-inflammatory effects of C5a binding occur through C5aR1,^40^ unchanged findings in C5aR1-deficient animals in this model could be possibly due to signaling through C5aR2.

Here, we showed a slight thickening of the C3aR1 KO mice retinal structure compared to WT mice 4 days after light exposure. This was mirrored by significant less MAC staining in the outer retina of C3aR1-deficient mice as well as less pronounced transcripts of complement proteins. Nevertheless, in situ detection of retinal cell death on cryosections of *C3ar1* mice via TUNEL did not indicate less apoptotic cell in KO mice compared to WT mice after 4 days. As retinal thinning is a gradual process, lower cell death levels comparing WT mice to KO mice could be expected at earlier time points. In fact, unpublished data of our group showed highest TUNEL^+^ cell counts 1 day after light exposure and only intermediate levels on day 4. Notably, also monocytes express C3a and C5a receptors, which could also influence retinal degeneration in this model. Furthermore, C3a stimulation of monocytes was shown to induce IL-1β secretion, subsequently to NLRP3 inflammasome activation,^45^ which would be a first line response in the tissue. In line with our data, C3aR1 was identified as a damaging neuroinflammatory factor in ocular hypertensive DBA/2J mice, influencing the microglial expression pattern and being associated with the risk of degeneration.^46^ Although drusen are a hallmark in the development of AMD and C3a trigger the formation of sub-RPE deposits in vitro,^47^ altered retinal structures in BAF fundus images could not be detected in our model.

Studies targeting complement in inflammatory mouse models yield diverse results. In the laser model of wet AMD neutralization of anaphylatoxins, or their respective receptors, resulted in a reduced neovascular area.^12^ In another choroidal neovascularization (CNV) study, antibody blocking of the alternative complement pathway with the fusion protein CR2-fH, combining the iC3b/C3d-binding region of CR2 and the N-terminus of the regulatory CFH, normalized anaphylatoxin levels, and reduced lesion size.^48^ Contrarily, blockade of the anaphylatoxin receptors was not sufficient to alter the course of lesion repair significantly in the same study.^48^ Dual time-dependent effects were demonstrates for C5aR1/C5a signaling in a spinal cord injury model^49^ as well as for C3aR signaling during ischemic injury of the adult brain.^50^ In a mouse model of experimental autoimmune uveitis (EAU) less severe uveitis in C3aR1/C5aR1-double KO mice than control mice was evident, involving reduced T cell response.^2^

Indeed, anaphylatoxin receptor signaling subsequently recruits immune cells and promotes the pro-inflammatory surrounding, which is considered the most important effect of complement dysregulation in AMD.^51^^,^^52^ Nevertheless, their signaling is also indispensable for tissue homeostasis and regeneration. Therefore, genetic compensatory mechanisms, as previously reported,^53^ due to full body KO used in this study, should be taken into account, when interpreting the study data. In vitro analyses of RPE cells suggest a combination of signaling between C3aR and C5aR1 in order to implement their precise immune regulatory functions.^54^ Future work will also be required to investigate the interplay among anaphylatoxin receptor signaling, complement activation, and MAC formation.

## Conclusions

Here, we tested full body KO mice of either *C3ar1* or *C5ar1* anaphylatoxin receptor in a mouse model mimicking some features of dry AMD. A partial rescue of retinal thickness was shown in C3aR1 KO mice, which was mirrored by a significantly less MAC occurrence in the outer retina. Furthermore, a tendency of a more regulated alternative complement pathway and inflammation was given. However, none of the tested conditions led to mitigated mononuclear phagocyte migration to the subretinal space. Whereas C3aR1-targeted therapy may be considered for patients with dry AMD, further work will be necessary to elucidate the optimal intervention point in the anaphylatoxin signaling axis. Furthermore, heterogeneity of disease progression may point to more patient-tailored therapy strategies, favoring from advanced technologies and imaging modalities.

## Supplementary Material

Supplement 1
