# Supplementary material for: C3aR1-Deletion Delays Retinal Degeneration in a White-Light Damage Mouse Model
Source: Invest Ophthalmol Vis Sci. 2025 Jan 7;66(1):15. doi: 10.1167/iovs.66.1.15 (PMC11717133; doi:10.1167/iovs.66.1.15)
Supplement: Supplement 1 [file iovs-66-1-15_s001.pdf]

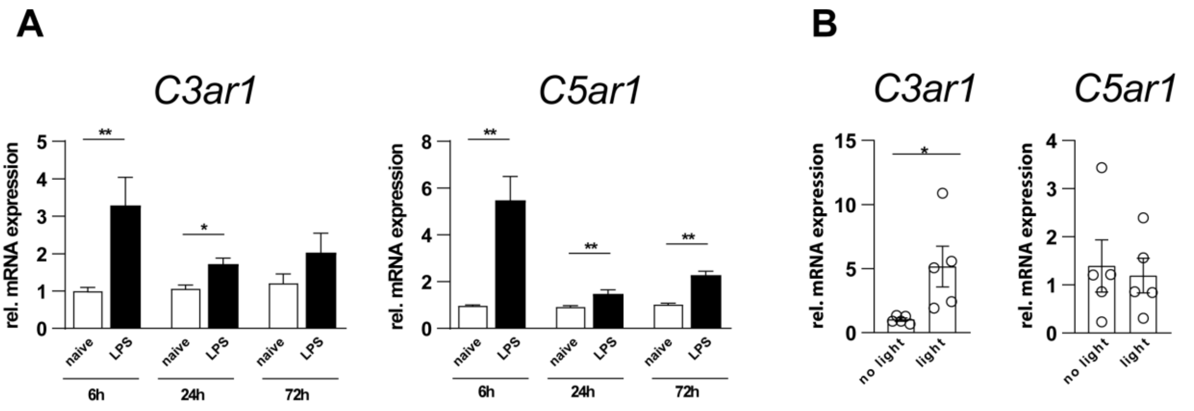

Supplementary Figure 1. Relative mRNA expression levels of *C3ar1* and *C5ar1* in BALBc mice and BV-2 microglia cells. **A** BV-2 cells were treated with 50 ng/ml Lipopolysaccharide (LPS) for 6, 24 or 72 hours (n = 6). **B** BALB/c mice were light damaged with 10,000 lux for 30 minutes; retinas were analyzed 4d after light damage (n = 5). qRT-PCR was performed and ΔΔCT analysis was used for quantification. *ATP5B* was used as reference gene. Graphs were plotted with GraphPad Prism 7.04. Bars represent Mean ± SEM. Data were analyzed using Mann Whitney t test.

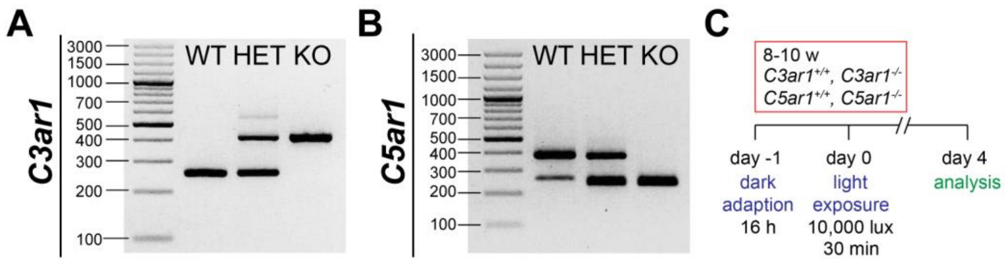

Supplementary Figure 2. Genotyping results and experimental setup. DNA was isolated from ear punches. **A** For *C3ar1* PCR samples were separated on a 2% agarose gel. Amplicons of 250 bp were considered WT and 400 bp KO. **B** For *C5ar1* mice PCR samples were separated on a 1% agarose gel. Amplicons of 386 bp were considered WT and 244 bp for KO. **C** 8-10-week-old male and female *C3ar1* or *C5ar1* WT and KO mice were dark adapted for 16 h before light exposure with 10,000 lux for 30 min and sacrificed for analysis 4d afterwards.

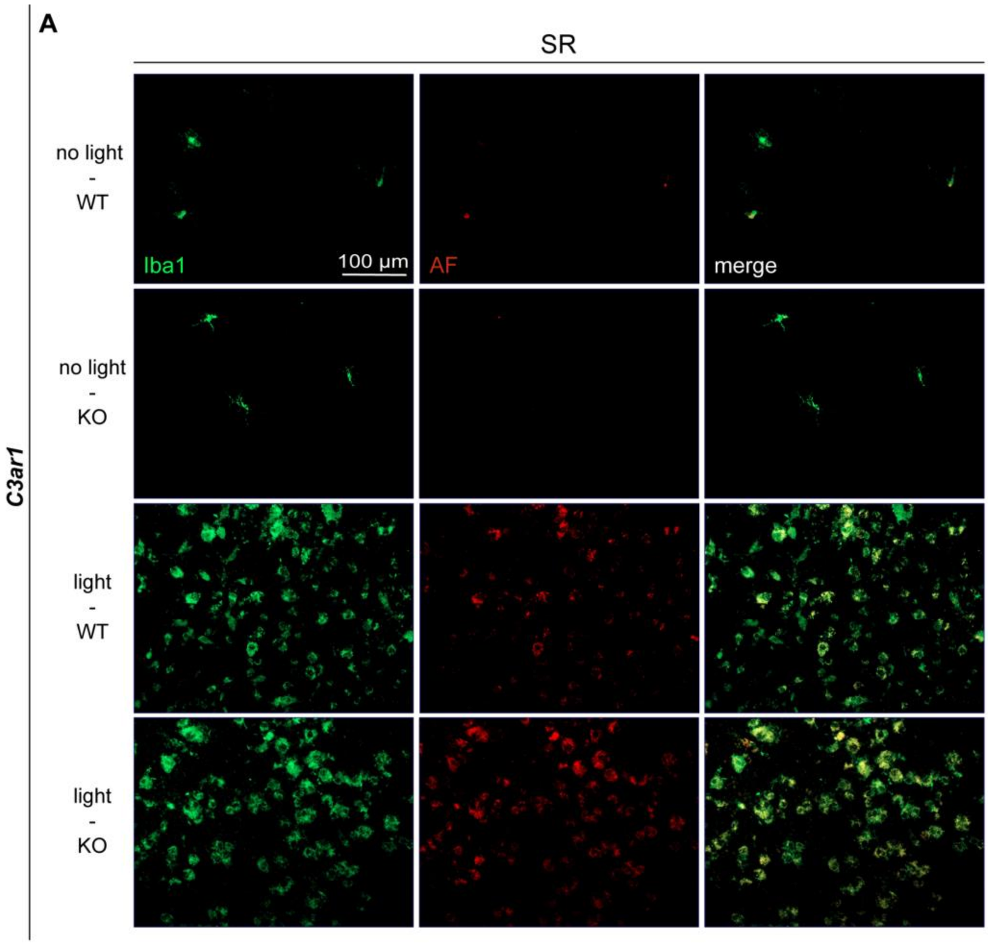
A
SR
no light
-
WT
no light
-
KO
light
-
WT
light
-
KO
C3ar1
Iba1
100 µm
AF
merge

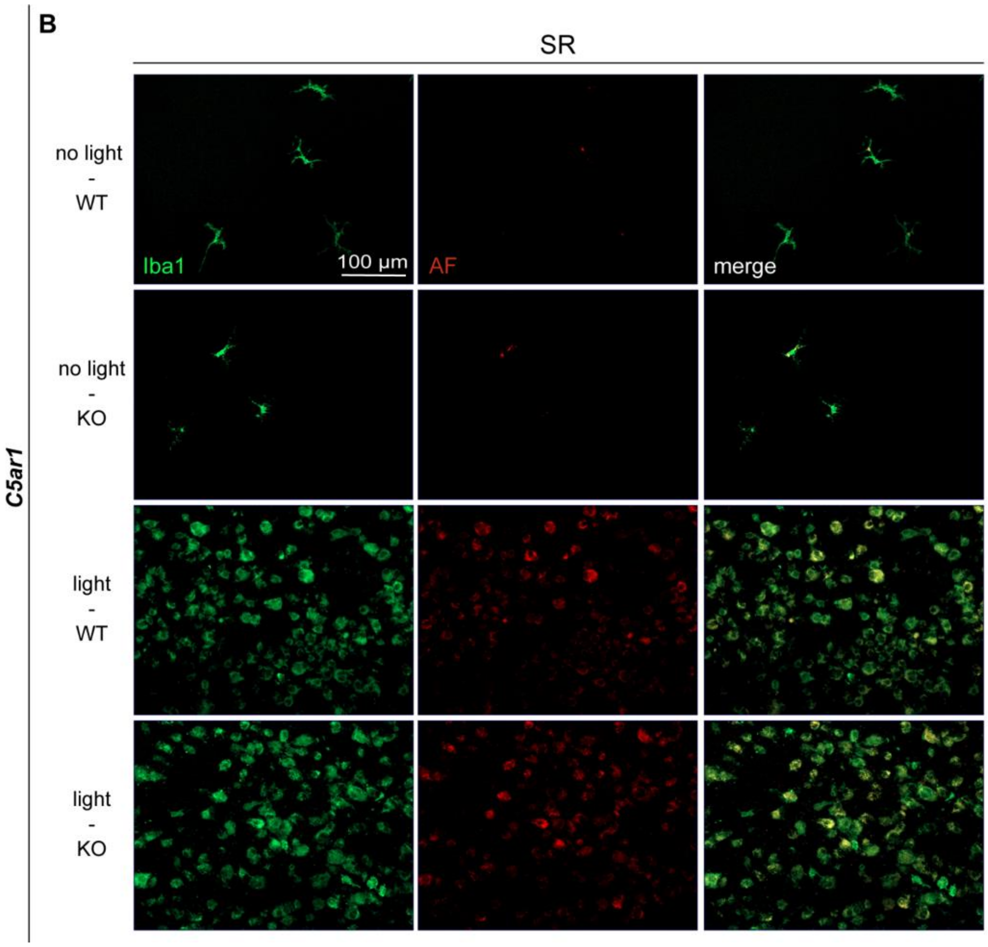
B
SR
no light
-
WT
no light
-
KO
light
-
WT
light
-
KO
C5ar1
Iba1
100 µm
AF
merge

Supplementary Figure 3. Single channels of morphological analyses of Iba1+ cells in the retina. Retinas were analyzed 4d after light damage with 10,000 lux for 30 minutes. **A, B** Mononuclear phagocytes were stained on retinal flat mounts against ionized calcium-binding adapter molecule 1 (Iba1, first column). Channel was merged (third column) with autofluorescence (AF, second column).

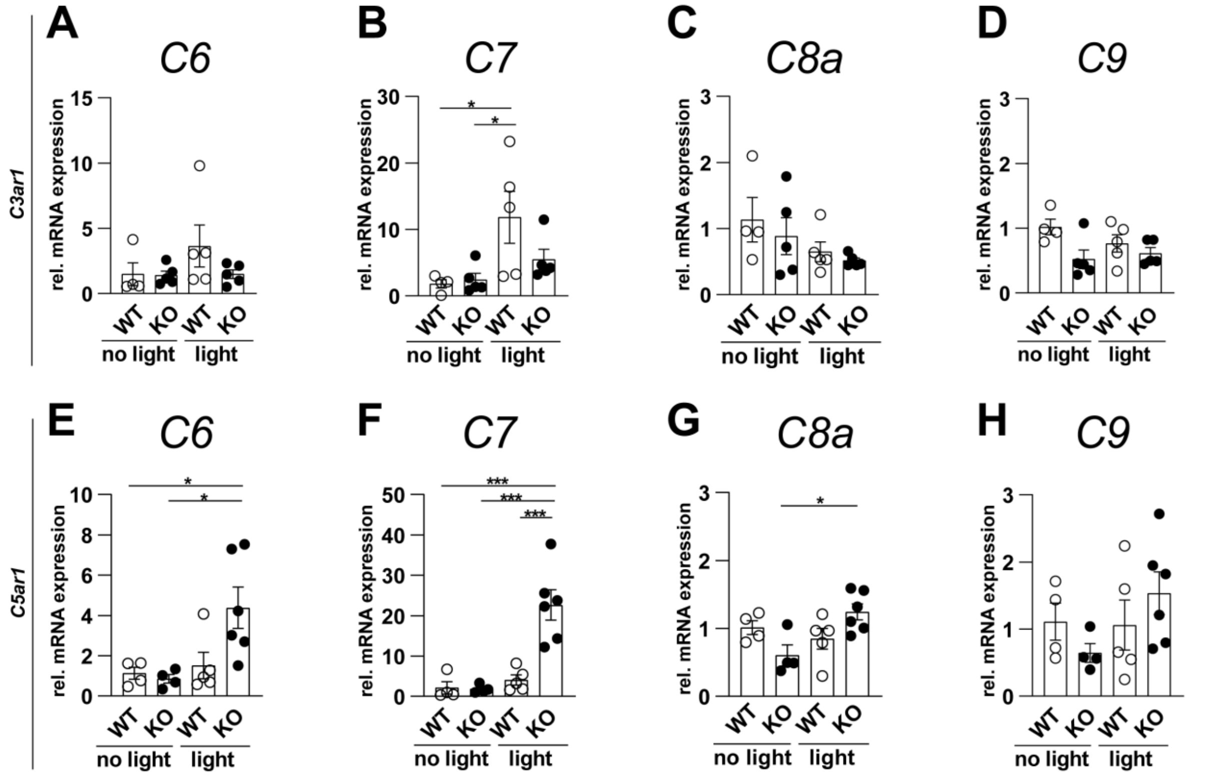

Supplementary Figure 4. Expression analysis of complement terminal pathway genes in retinal tissue. Retinas were analyzed 4d after light damage with 10,000 lux for 30 minutes. qRT-PCR was performed and ΔΔCT analysis was used for quantification. *ATP5B* was used as reference gene. Graphs were plotted with GraphPad Prism 7.04. Bars represent Mean ± SEM. Data were analyzed using one-way ANOVA followed by Tukey´s multiple comparison post-test (n =11-15).
